# Progress of Epidemics in Forty-Two Towns and Districts

**Published:** 1858-10

**Authors:** 


### PROGRESS OF EPIDEMICS.

### LOCAL REPORTS OF EPIDEMIC AND ENDEMIC DISEASES

*During the Months of June, July, and August*, 1858.

| Place. | County. | Lat. | Long. | Observer. |
|---|---|---|---|---|
| St.Mary, Scilly | Cornwall - | 49.50 N. | 6.24 W. | J. G. Moyle, Esq. |
| Teignmouth - | Devonshire - | 50.32 N. | 3.29 W. | W. C. Lake, Esq. |
| Portsmouth - | Hampshire - | 40.58 N. | 6. 6 W. | Alfred Jackson, M.D. |
| Odiham - - | Hampshire - | 51. 8 N. | 1. 3 W. | J. McIntyre, M.D. |
| Canterbury - | Kent - - | 51.17 N. | 1. 4 E. | G. Rigden, Esq. |
| Chatham - | Kent - - | 51.21 N. | 0.14 E. | F. J. Brown, M.D. |
| Wandsworth - | Surrey - - | 51.28 N. | 0. 7 W. | G. E. Nicholas, Esq. |
| Putney - - | Surrey - - | 51.28 N. | 0. 8 W. | R. H. Whiteman, Esq. |
| Up. Holloway | Middlesex - | 51.32 N. | 0.03 E. | W. B. Kesteven, Esq. |
| Hawkesbury - | Gloucestersh. | 51.34 N. | 3.20 W. | W. I. Cox, Esq. |
| Swansea - | Glamorgansh. | 51.38 N. | 2.50 W. | W. H. Michael, Esq. |
| Colchester - | Essex - - | 51.54 N. | 0.52 E. | Henry Laver, Esq. |
| Aspley Guise - | Bedfordshire - | 52. 1 N. | 0.37 W. | J. Williams, M.D. |
| Saffron Walden | Essex - - | 52. 3 N. | 0.12 E. | T. Spurgin, Esq.<br>H. Stear, Esq. |
| Bedford - - | Bedfordshire - | 52. 8 N. | 0.51 W. | T. H. Barker, M.D. |
| Newpt.Pagnell | Buckinghams. | 52.10 N. | 0.42 W. | G. O. Rogers, Esq. |
| Sharnbrook - | Bedfordshire - | 52.12 N. | 0.40 W. | R. S. Stedman, Esq. |
| Wellingbro' - | Northamptons. | 52.20 N. | 0.40 W. | B. Dulley, Esq. |
| Beccles - - | Suffolk - - | 52.25 N. | 1.48 E. | W. E. Crowfoot, Esq. |
| Thetford - | Norfolk - - | 52.26 N. | 0.45 E. | H. W. Bailey, Esq. |
| Stourbridge - | Worcestersh. - | 52.28 N. | 2.32 W. | A. Freer, Esq. |
| Dudley - - | Staffordshire - | 52.30 N. | 2.10 W. | J. H. Houghton, Esq. |
| Barrowden - | Rutland - | 52.34 N. | 0.38 W. | H. J. Swann, Esq. |
| Wisbeach - | Cambridgesh. | 52.39 N. | 0. 5 E. | W. H. Hole, Esq. |
| East Dereham | Norfolk - - | 52.40 N. | 0.57 E. | J. Vincent, M.D. |
| Pontesbury - | Shropshire - | 52.43 N. | 2.50 W. | Wm. Eddowes, Esq. |
| Nottingham - | Nottinghamsh. | 52.50 N. | 1.10 W. | T. Robertson, M.D. |
| Burton-on-Trt. | Staffordshire - | 52.53 N. | 1.53 W. | S. Thomson, M.D. |
| Oswestry - | Shropshire - | 52.53 N. | 2.59 W. | P. Cartwright, Esq. |
| Wrexham - | Denbighshire - | 53. 2 N. | 3. 1 W. | E. Williams, M.D. |
| Hawarden - | Flintshire - | 53.11 N. | 3. 2 W. | T. Moffat, M.D. |
| Lincoln - | Lincolnshire - | 53.12 N. | 0. 5 W. | S. Lowe, Esq. |
| Alford - - | Lincolnshire - | 53.15 N. | 0. 6 E. | R. U. West, M.D. |
| Gainsborough | Lincolnshire - | 53.23 N. | 0.47 W. | D. Mackinder, M.D. |
| Liverpool - | Lancashire - | 53.24 N. | 2.59 W. | Thos. Bickerton, Esq. |
| Warrington - | Lancashire - | 53.25 N. | 2.32 W. | C. N. Spinks, Esq. |
| Wigan - - | Lancashire - | 53.32 N. | 2.33 W. | Jephtha Hussey, Esq. |
| Bolton - - | Lancashire - | 53.35 N. | 2.19 W. | W. H. Pendlebury, Esq. |
| York - - | Yorkshire - | 53.58 N. | 1. 3 W. | W. Procter, Esq. |
| Wst. Auckland | Durham - | 54.45 N. | 1.40 W. | G. Todd, Esq. |
| Rothbury - | Northumberld. | 55.25 N. | 1.50 W. | E. C. Summers, Esq. |
| Lerwick - | Shetland Isles | 60. 9 N. | 1. 8 W. | G. W. Spence, M.D. |

### QUARTERLY STATEMENT—No. XV.

[*The dates denote that the disease appeared in the weeks then ending.*]

SCARLET FEVER.

Portsmouth—June 11
Odiham—June 11-25, July 30, all Aug.
Canterbury—All August
Chatham—All June, July 2, 16, 30
Putney—June 4, August 20
Up. Holloway—August 13-27
Swansea—June 4, 18, July 2
Colchester—Every week
Aspley Guise—June 18-25, July 2*

* One case only, imported from a neighbouring village.

Beccles—June 4-11, July 23-30, all Aug.
Thetford—All August
Dudley—June 18-25, July 2, Aug. 6-13
Barrowden—All August
Wisbeach—August 13-27
East Dereham—August 13-27
Pontesbury—June 18-25, all July & Aug.
Nottingham—July 23-30
Burton-on-Trent—June 4-11, July 23
Lincoln—All July
Alford—August 20
Gainsborough—June 25, July 2-16, Aug. [13
Liverpool—Every week
Warrington—August 13, 27
Wigan—July 2, 23, August 27
Bolton-le-Moors—All June, July 2-9, 30, all August
York—June 18-25, July 9-30, all Aug.

MEASLES.

Portsmouth—June 4
Odiham—July 23, August 20-27
Canterbury—Every week
Chatham—All June, July 2, 23, Aug. 6
Wandsworth—July 30, Aug. 6-20
Swansea—Every week
Colchester—June 11-25, July 16-30, [Aug. 6
Bedford—All August
Beccles—All June, July 2
Stourbridge—July 16-30, Aug. 27
Dudley—June 11-25, July 9-23, Aug. 13- [27
Alford—June 11, 25, all July & Aug.
Gainsborough—June 25
Liverpool—Every week
Warrington—July 2-16
Wigan—June 11-18, July 9, 23, Aug. 6, 27
Bolton-le-Moors—June 11-25, July 2, 23-30, all August
York—June 18, July 2, 23, Aug. 6-20
Lerwick—August 6, 20

SMALL-POX.

Portsmouth—June 11-18, July 30
Canterbury—July 23-30, Aug. 6-13
Chatham—All June and July, Aug. 6
Up. Holloway—July 9
Hawkesbury Upton—June 4-18
Swansea—June 4-18
Colchester—June 25, July 2-9, 30, Aug. 6
Bedford—August 27
Stourbridge—June 4, 18-25, July 9
Dudley—June 25, July 16-30, Aug. 13, 27
Barrowden—June 4, 18
Wisbeach—June 4-11
Pontesbury—July 9-30
Nottingham—June 4
Burton-on-Trent—June 4, 25
Oswestry—All June, July 2-16
Wrexham—Every week
Gainsborough—July 30, all August
Liverpool—Every week
Wigan—June 11, July 16
Bolton-le-Moors—Every week
Lerwick—June 4, 18

HOOPING COUGH.

St. Mary's, Scilly—All June, July 2-16
Portsmouth—July 9
Canterbury—June 4-18, July 9, 23
Chatham—July 9
Wandsworth—All June, July 9, 23-30, [Aug. 13
Up. Holloway—All June
Hawkesbury Upton—All June & July, [Aug. 6-13
Swansea—Every week
Colchester—Every week
Bedford—July 16-30, all August
Newport Pagnell—July 2, Aug. 27
Beccles—June 4-18
Thetford—August 20
Dudley—June 11, July 9
Wisbeach—June 4-18
Pontesbury—Every week
Nottingham—June 4-18
Burton-on-Trent—July 2, 30, Aug. 6-13
Hawarden—June 18
Alford—Every week
Gainsborough—Every week
Liverpool—Every week
Warrington—June 11-18, July 16, 23
Wigan—June 11-18, July 9, 23, Aug. 6, 27
Bolton-le-Moors—June 11-25, July 23-30, August 6, 20-27
York—June 4, 18, July 2, 16
Rothbury—July 16

CROUP.

St. Mary's, Scilly—August 20
Portsmouth—August 27
Chatham—July 16
Hawkesbury Upton—June 4
Swansea—June 11-25, July 9, 23
Newport Pagnell—August 20
Beccles—August 20-27
Thetford—June 4
Stourbridge—August 6-13
Dudley—July 23
Liverpool—June 11, 25, July 2, 16, Aug. [6, 27
Wigan—August 6
Bolton-le-Moors—June 18-25, July 16- [23, Aug. 6
York—July 9, Aug. 6

CATARRH.

Teignmouth—July 16
Odiham—June 4-18, July 9-23, Aug. 13- [27
Chatham—June 11-18, July 2, 30, Aug. 20-27
Wandsworth—June 4, 18, July 2, 23-30, August 13-27
Putney—June 4-11, 25, July 2-9, 23-30, [Aug. 6-20
Up. Holloway—June 18-25, July 2
Hawkesbury Upton—August 20-27
Swansea—June 4-18
Saffron Walden—June 25
Newport Pagnell—June 4-11, Aug. 20
Beccles—June 11-18, July 2 23

Thetford—June 4, 18-25, July 9-16, August 6-13, 27
Dudley—June 18, July 16, Aug. 13
Wisbeach—August 13-27
Nottingham—June 4
Oswestry—Every week
Gainsborough—All June, July 2-30, [Aug. 6-20
Liverpool—Every week
Bolton-le-Moors—June 4-18, July 16-30, August 13-20
Lerwick—June 4, 25, July 2-9, Aug. 6

INFLUENZA.

Chatham—June 4, July 2, Aug. 13 27
Aspley Guise—July 9-30
Newport Pagnell—All June & July, Aug. [20
Sharnbrook—June 11-18
Beccles—June 4-11
Thetford—June 4-11, 25, July 9, 30, [Aug. 6, 20-27
Oswestry—July 9-23
Liverpool—All June, July 2-23
Bolton-le-Moors—June 18-25, July 2, 30, August 6-13
York—June 4, 18-25, July 9, 23
Lerwick—June 4

ERYSIPELAS.

Portsmouth—June 11
Odiham—August 6, 20-27
Canterbury—July 9
Chatham—June 4, July 2, all Aug.
Wandsworth—June 11, July 30, Aug. 13, [27
Putney—June 11-18
Saffron Walden—August 13, 27
Bedford—June 25, July 2
Newport Pagnell—July 30, Aug. 6, 27
Sharnbrook—July 9, 23
Thetford—June 4, 18-25, July 16, Aug. 27
Stourbridge—July 30, August 6
Dudley—June 18, July 2-9, Aug. 20
Wisbeach—All August
East Dereham—August 13-27
Pontesbury—July 23-30, Aug. 6-20
Nottingham—July 2, August 13
Burton-on-Trent—June 4, July 23
Oswestry—July 23-30, Aug. 6-13
Wrexham—August 6-13
Alford—July 9, August 6
Gainsborough—July 23, Aug. 27
Liverpool—Every week
Bolton-le-Moors—June 11-25, July 2, 23-30, August 6, 27
Lerwick—June 11

CHOLERA.

Chatham—July 2, 16, 30, Aug. 6
Alford—August 20-27
Bolton-le-Moors—June 4-18, July 9-30

AGUE.

Southsea—June 11
Odiham—July 23
Canterbury—June 18-25, Aug. 13
Chatham—All June and July, Aug. 27
Colchester—Every week
Bedford—All June
Beccles—August 13-27
Thetford—June 4-11, 25, July 16-23, August 6, 20-27
Barrowden—June 4-18, July 2-9, 23-30
Wisbeach—All June, July 2-16
East Dereham—Every week
Nottingham—June 4, 18, July 9
Burton-on-Trent—July 23
Lincoln—All June
Alford—All June

REMITTENT FEVER.

Teignmouth—June 11-18, July 9, 30, August 6-13, 27
Canterbury—June 11-18, July 2
Putney—June 4, July 30
Saffron Walden—June 4
Newport Pagnell—June 4-11, July 30, August 13, 27
Sharnbrook—July 23-30, all Aug.
Beccles—June 25, July 2-9
Thetford—June 25. July 16-23
Dudley—June 11
Nottingham—July 2, 23-30, Aug. 6
Oswestry—June 25, July 2-9, Aug. 13-27
Alford—June 11-25, all July and Aug.
Warrington—July 16
Bolton-le Moors—Every week
York—June 11, July 2, 23, Aug. 20
Rothbury—June 11-18, August 6

DIARRHŒA.

St. Mary's, Scilly—August 13, 20
Teignmouth—June 11, 25, July 9, 30, August 6-13, 27
Southsea—July 16, 30, Aug. 6-20
Odiham—All August
Canterbury—June 25, all July & Aug.
Chatham—All June, July 2-9, 23-30, all August
Wandsworth—June 4, 18-35, all July & [Aug.
Putney—June 25, July 2 23, Aug. 6
Up. Holloway—June 18-25, July 2, 23, August 27
Hawkesbury Upton—Aug. 13-27
Swansea—Every week
Colchester—July 30, all August
Aspley Guise—July 23-30, Aug. 6
Saffron Walden—June 11, 25, July 23-30, August 6, 20
Bedford—June 18, July 2, 16, 23, all Aug.
Newport Pagnell—Every week
Sharnbrook—June 11, July 2-23, Aug. 13-27
Wellingborough—All June, July 2, Aug. [13-27
Beccles—June 18, Aug. 13-27
Thetford—Every week
Stourbridge—July 23-30, all Aug.
Dudley—June 11-25, all July & Aug.
Barrowden—June 11, July 2, 30, Aug. 20

Wisbeach—All June, July 2-16, Aug. 20-27
East Dereham—July 16-30, all Aug.
Pontesbury—All July and August
Nottingham—June 4, July 2
Burton-on-Trent—July 16, Aug. 20-27
Oswestry—Every week
Wrexham—August 13-27 [6,20
Hawarden—June 4,25, July 16, 30, Aug.
Lincoln—July 9-30, August 6-20
Alford—June 25, July 2, 23-30, all Aug.
Gainsborough—Every week
Liverpool—Every week
Warrington—August 13-27
Wigan—June 11-25, July 9, Aug. 6, 20, 27
Bolton-le-Moors—June 11-25, July 9-30, all August
York—June 18, July 9-30, all Aug.
Rothbury—June 4, 25, July 2-9
Lerwick—June 4, July 2-9, Aug. 6

DYSENTERY.

Teignmouth—July 2-9
Odiham—August 20-27
Chatham—June 4, 25, July 9-16
Wandsworth—June 11, July 2-9
Up. Holloway—July 16
Newport Pagnell—All July, Aug. 20
Stourbridge—July 30, Aug. 6, 27
East Dereham—July 16-30
Nottingham—June 11, July 9-16, Aug. 6
Hawarden—August 6
Alford—June 25, July 2
Gainsborough—June 18-25, July 2, 23-
Liverpool—Every week [30
Wigan—August 27 [Aug. 6-13
Bolton-le-Moors—All June, July 16-30,
York—July 9, 23, August 6-20

TYPHUS.

Teignmouth—June 11, July 2, 16-30, August 6-13
Portsmouth—June 25, July 9-30, Aug.
Odiham—All August [20
Canterbury—Every week [27
Chatham—June 11-18, July 30, Aug. 20-
Wandsworth—All June, July 2 9, 23-30, August 6-13, 27
Putney—June 4, August 27
Bedford—June 11, 25, July 2-16, 30
Thetford—June 4, all August
Dudley—June 11-25, all July & Aug.
Wisbeach—August 13-27
NottinghamJune 25, August 13
Wrexham—All August
Lincoln—August 6 20
Alford—June 25, all July and Aug.
Gainsborough—August 13
Liverpool—Every week
Bolton-le-Moors—June 11-25, July 23-30, August 13-27

PUERPERAL FEVER.

Alford—June 4 [6
Bolton-le-Moors—June 25, July 2, Aug.

DIPHTHERITE.

Portsmouth—June 25, July 2-9, Aug. 27
Canterbury—June 25, July 2-30
Thetford—July 9, 30, Aug. 6-13, 27
Lincoln—July 16-30, August 6-20
Alford—July 30, all August

TYPHOID FEVER.

Colchester—Every week
Stourbridge—August 20-27
Burton on-Trent—August 20-27

RHEUMATISM.

Thetford—June 4-11, 25, July 9-23, August 6-20

CARBUNCLE.

Portsmouth—June 25, Aug. 13
Chatham—July 16-30
Swansea—July 23-30, all August
Saffron Walden—August 6, 20
Newport Pagnell—July 30, Aug. 6
Sharnbrook—August 13-27
Beccles—July 16-23, Aug. 20-27
Stourbridge—August 6
East Dereham—August 20-27
Pontesbury—June 25
Nottingham—June 18
Oswestry—July 2-16
Wrexham—August 13-20
Gainsborough—August 20-27
Bolton-le-Moors—June 18-25, July 30

---

### ADDITIONAL OBSERVATIONS.

*St. Mary's, Scilly.*—Mr. Moyle says: "This quarter, ending August 27th, has been, on the whole, remarkably healthy. Hooping-cough continued on the Islands until the third week in July. There was one death from hooping-cough, complicated with dentition and epilepsy, in a child of eight months. On August 9th, there was one death from croup; the first case I had witnessed during my residence at Scilly (nine years). I was called late to the case, and could not induce the parents to consent to tracheotomy. I do not know to what to attribute this freedom from croup in these Isles of the Atlantic, unless it be our equable temperature. The average ozone for the quarter has been 3.1 per week. The weather has been very fine on the whole; the depth of rain for the quarter being 6.8 inches. The highest day temperature was 79°, the lowest night temperature 54° Fahr. The harvest has been an average one; and there has been no disease among the cattle."

*Teignmouth.*—Mr. Lake writes: "The temperature of the air, which had risen rather suddenly at the end of May, continued remarkably high through June. It fell a good deal during the first ten days of July, rose during the middle of the month, fell somewhat at its close, again rose, and then declined during the latter half of August. The highest points reached during these periods were, 78.0° on June 23rd; 78.6° on July 12th; and 78.7° on August 10th. The mean temperature of July (60.1°) and of August (61.1°) was about the average; but June was almost uninterruptedly a period of elevated temperature. Its mean temperature (61.4) was 3.6° above its average for the last five years, while its mean maximum temperature (72.0°) was above that of any month of the last five years, with the exception of August 1857. The mean barometric pressure of June was 30.035; that of July, 29.919; of August, 29.955. The air during the quarter was on the whole dry. The rain fall, with the exception of that on one day, not very great. Of the 4.897 inches that fell during the three months, 2.087 fell on July 27th. The wind during each month blew most frequently from the north. The amount of ozone for June was 5.9; for July, 6.4; for August, 6.0. A good deal of thunder and lightning occurred between the 7th and 10th of June. The town, during the quarter, has been very healthy. Disorders affecting the respiratory organs have been very much in abeyance; whilst those affecting the digestive system have not been numerous. Thirteen cases of diarrhœa only have come under my notice in the three months, five of which occurred in infants; and only two of dysentery. Nine cases of fever in adults have come before me in the same time; and although, in the list placed under the head of typhus, they have no more resemblance to the typhus of Jenner, or the old-fashioned low typhus fever of books, than they have to scarlatina, their type being the bilious or gastric, with a tendency to remission; the usual type, indeed, of fever here."

*Southsea.*—Dr. Jackson writes: "Two children were attacked with diphtheria in one house—a boy, aged 6, and a girl, aged 3 years. There was more local mischief in the boy's case; and a large leather-like mass was discharged from the fauces. He recovered. The girl was under treatment much earlier; there was less local mischief, but more constitutional disturbance. She died. The mother and nurse had small white patches on the fauces, and also a servant, who was borrowed for the occasion. Two other servants, and the father, who did not attend to the child, escaped. In another house, three adults had slight sore throat, one showing small white patches. The treatment consisted in frequent application of dilute hydrochloric acid, tincture of sesquichloride of iron, emetics, wine, etc."

*Canterbury.*—During the past quarter, in a population, according to the last census, of 18,398 inhabitants, there have been twenty-four deaths from zymotic diseases; viz., eleven in June; eight in July; and five in August. Of these deaths, two were caused by hooping-cough; six by fever; four from diphtheria; five from measles; two from scarlatina; two from diarrhœa; one from syphilis, an infant three weeks old; and two from variola, aged respectively six weeks and two years, both unvaccinated.

*Chatham.*—Dr. Brown writes: "Stomatitis and diphtheria have been very prevalent. Small-pox has occurred as an epidemic of some severity. Idiopathic peritonitis has killed several persons during the past quarter. The average duration of the disease was four days. There have been a few cases of phlegmasia dolens, but none of metria. Nephritis was prevalent in June. A man died in the week ending June 25th, of spinal meningitis of five days duration, and of purpura of two days duration. His illness was attributable to insolation. A woman died in the week ending August 27th, of tetanus, of sixty-one hours duration. She had old ulcers about the knee. The cause of the attack was unknown."

*Putney.*—Mr. Whiteman writes: "The past quarter has been characterised by the prevalence of diarrhœa to some considerable extent, though no cases of very great severity came under treatment. Scarlatina, which had all but disappeared at the commencement of the quarter, and was absent during nearly the whole of June, July and August, again showed itself at the termination of the last named month, and still continues. Several cases of malignant sore throat in young children occurred during the quarter, but the malady was confined to one family, and to one house at Roehampton, a very dry and elevated locality. Two of the children first attacked died within a very short time of the invasion of the disease. In each of these two fatal cases there was noticed an abscess in the pharynx, and in the first case it was so certified as the immediate cause of death by the surgeon who attended. All the other cases (five in number) came under my own observation and treatment, and in each of them I noticed, besides a highly congested state of the fauces, sloughing of the tonsils and some ulcerative patches about the mouth. No eruption of any kind, however, was observed either to precede or accompany the disease. In the treatment of the four children who recovered, the nitrous acid gargle was employed with excellent effect. The mortality of this parish during the past quarter was very considerably below the average."

*Hawkesbury Upton.*—Mr. Cox writes: "The small-pox was brought here during May by a visitor from Bath, where the epidemic was very prevalent. The cases here were happily limited to five; all in one house. The greatest precautions were successfully taken to prevent its spread. One of the cases was that of an infant, aged ten months, who had been vaccinated four days before the onset (rigor and sickness) of the variola. The vaccine vesicle was, nevertheless, fully developed, and the two diseases ran their course for a certain time together. None of the cases proved fatal. Hooping-cough has been the most prevalent epidemic during the quarter. I have had fifty-two cases under treatment; three of which proved fatal, from the supervention of pneumonia. The cattle and sheep in this district have been quite free from epizootic maladies. Vegetation has been generally healthy, and the potatoe disease has not yet visited us. The temperature during June 12th to 22nd, and August 14th to 26th, was very high."

*Colchester.*—Mr. Laver says: "Scarlatina has been prevalent this quarter; but most of the cases have been slight. Small-pox has occurred in a few isolated cases: I have not heard of any fatal results. Typhoid fever has been prevalent, but not to so great an extent as in the corresponding quarter of last year. There have been a few cases of diphtheria. Altogether this quarter has been unusually healthy. Potatoes are much diseased; otherwise vegetation is healthy."

*Aspley Guise.*—Dr. Williams says: "The past quarter has been very healthy generally; and, though June was two degrees above the average of several years, July two degrees, and August one degree below the average, the weather was very stormy and changeable. As a consequence, catarrh, and one bad case of influenza, ensued. There have also been a number of slight cases of simple cynanche tonsillaris, evidencing the same epidemic influences, modified by a dry healthy locality; but no real case of diphtherite occurred, though it seems to have prevailed severely in many parts of England. Two cases at Ridgemount, of a modified character, ended favourably. They originated within a few yards of the spot where cholera broke out in 1854. The locality in question is entirely undrained, and infiltrated with the soak of the village, which in a porous soil gives off very unwholesome exhalations during hot weather. Added to this, the sleeping rooms thereabout are very small and insufficiently ventilated; over-crowding exists to an improper and dangerous extent."

*Saffron Walden.*—Mr. Stear says that the quarter was very healthy; but pleurisy and pneumonia were as prevalent as in winter, depending upon the great and sudden alternations of temperature in the district during the quarter.

*Wellingborough.*—Mr. Dulley states that this neighbourhood has been singularly free from illness of any kind.

*Thetford.*—Mr. Bailey sends the following report of diseases for the quarter, ending August 27:—"June. We have not experienced for many years so uniform a state of high temperature as in this month; the maximum being on the 16th, at 90°, and the evenings seldom lower than 65°. The barometrical range deviated very little, which may be attributed to the absence of wind. The fall of rain was only 0·3 of an inch; and the mean of dew-point throughout the month as high as 60°. Distant thunder was heard on the 9th, and only one thunder-storm was experienced on the 17th, when the greatest quantity of rain fell. The nights were also very oppressive, oftentimes as high as 74°. During the last week, a sudden change was felt, by the temperature falling in the middle of the day to 74°; and at night, on the grass, to 39°. To the little deviation of temperature and atmospheric pressure, we may attribute the few diseases which came under notice in this month, depending upon atmospheric influence. No specific epidemic occurred. A solitary case of croup in a boy of six years of age came under treatment; but he ultimately recovered. Catarrhs and influenza were more prevalent, attacking elderly people. Erysipelas has been more general the last year than in any previous year; during this month, five cases came under medical treatment, and in two instances seemed contagious, During the greatest heat of the month, two cases of ague were taken to the union workhouse, of labouring men, who worked late in the evenings; they soon recovered. One case of typhus occurred in a labouring man of intemperate habits; it ran a long course and ultimately recovered, leaving the greatest prostration of strength. With the casual cases, a very severe case of hæmorrhagic purpura came under notice with hæmoptysis: the whole body was covered with petechiæ. Rheumatism, diarrhœa, hepatitis, cynanche tonsillaris, paralysis, and hysteria, were observed.

"July. The thermometrical range was that of a summer month, ranging between 60° and 80°, with the exception of two days, when it rose to 84° and 86°. From the wind being generally in the north, with frequent showers during the month, the temperature did not exceed 70°. The atmospheric pressure was more fluctuating than last month, from the frequent winds and showery weather. The quantity of rain fallen in the month was 243 inches; and the dew point was 57°. But few diseases occurred during the month; no epidemic. Catarrhs were still prevalent; and only one case of influenza occurred in an old lady, and erysipelas of a slight kind occurred in a girl; she had been subject to it from any slight cause. Diarrhœa was more prevalent, attacking children and adults in some cases very severely. Rheumatism and bronchitis were under treatment; and one case of diphtheria occurred in a child. The casual complaints under care, were anasarca, hepatitis, pleurodynia, carditis, amenorrhœa, urethal stricture, hysteria, cystorrhœa, lumbago, and nephralgia.

"August. During the first half of the month, we had a high temperature ranging between 70° and 86°; and the mean of the dew point between 53° and 64°. Some sudden deviation of temperature took place, which I have observed for a series of years to occur when the planet Herschel has been in aspect or conjunction with other planets. In this month, it was remarkably so. On the 15th and 16th, the temperature was reduced 12° to 14° under such planetary aspect; after which it rose on the 18th to 81°, and the dew point to 64°. In the evening a most terrific thunder-storm passed over us from the east, lasting three hours, and accompanied by half an inch of rain. From this time to the end of the month, the temperature became cooler, ranging between 55° to 68°. This sudden change gave us an increase of catarrhal affections, and influenza became more prevalent. Bronchitis in children was also more general. Scarlatina still continued, attacking several children, and those convalescent, from exposure were affected with glandular anasarcous swellings. Several cases of diphtheritis came under medical care, occurring in children, the sloughs being extensive and the fœtor great. One death took place in a child three years of age. Ague in the working-men increased, especially in those who had been at harvest in the fens; and pleurodynia also. Diarrhœa was, especially the last week, very prevalent, attacking all grades and producing extreme prostration in children; some deaths have occurred. For some years I have not known typhus so frequent at this season of the year, not confined to the labourer, but attacking those of high station. It commenced with diarrhœa, much fever, brown tongue, and the greatest debility I ever witnessed. It runs a long course, and a slow recovery takes place. Croup attacked a child the last week in this month, from imprudent exposure late in the evening; and its recovery is doubtful.

"I find but little disease among the cattle. The sheep are particularly healthy. Vegetation has been very forward. Potatoes are less diseased. The harvest is finished in this locality."

*Record of Deaths in Twenty-one Parishes* (*population* 9,574) *from the Registrar's Book.*

| *June.* | | *July.* | | *August.* | |
|---|---|---|---|---|---|
| Consumption | 2 | Consumption | 2 | Typhus | 1 |
| Pneumonia | 1 | Diseased heart | 1 | Scarlatina | 2 |
| Diseased heart | 1 | Paralysis | 1 | Diseased heart | 2 |
| Hemiplegia | 1 | Convulsions | 2 | Convulsions | 1 |
| Cancer uteri | 2 | Diarrhœa | 1 | Diseased liver | 1 |
| Dropsy | 1 | Exhaustion from labor | 1 | Atrophy | 1 |
| Debility | 1 | Dropsy | 1 | Debility | 2 |
| Decay of nature | 2 | Debility | 3 | Decay of nature | 2 |
| Accident | 1 | Decay of nature | 6 | | |
| Suicide | 1 | Accident (rup. of blad.) | 1 | | |
| | 13 | | 19 | | 12 |

*Barrowden.*—Mr. Swann writes: "In June, the weather was exceedingly hot; July came in very cold; the latter part, and the first part of August, were excessively hot. A few cases of diarrhœa occurred, but nothing in comparison to the same quarter of last year. The prevailing disease was ague, which occurred to an extent previously unknown in this neighbourhood. Small-pox has left this district. Scarlet fever has broken out again at North Luffenham in a very malignant form. I do not hear of the potatoes being diseased at present."

*East Dereham.*—Dr. Vincent can only account for the number of cases of ague (here a very rare disease) by the drying up of the various ponds in consequence of the long continued drought.

*Pontesbury.*—Mr. Eddowes writes: "The only case of small-pox in this neighbourhood, during the present quarter, was imported from Bath. Capt. ——, Royal Navy, and his wife, came here on a visit to his uncle, the Rev. ——. He had been here just a week when he was seized with it; he had it severely, but recovered. I vaccinated his uncle, who is in his 71st year; he had had small-pox upwards of sixty years ago by inoculation: the cicatrices were quite visible. The vaccination took effect, but there was not a genuine vaccine vesicle. It was six weeks before it healed up. The skin of both hands and feet exfoliated. I revaccinated his niece and all the servants, and the Captain's wife; some of them took effect, others did not. They all escaped the small-pox. At the same time I vaccinated the Rev. ——; he had had small-pox in the natural way upwards of fifty years ago. His took effect; he had a genuine vaccine vesicle, which went through the regular stages. Seven weeks elapsed before the scabs fell off; the cicatrices are very distinct."

*Burton-on-Trent.*—Dr. Thomson writes: "Small-pox, after occurring in a series of scattered cases for some months, has completely disappeared from my country district; no fatal case having presented itself. The case of ague reported was a mild tertian, occurring in a lady who had formerly suffered from the disease when resident in Lincolnshire, and who, after three years absence, had revisited her old place of abode. The disease manifested itself after her return to this neighbourhood, but quickly yielded. Towards the end of the quarter diarrhœa and typhoid fever have become prevalent, especially since abundant moisture has succeeded a long period of drought. Mushrooms are more abundant than for some years past."

*Hawarden.*—Dr. Moffat writes that potatoe disease was first observed on June 27th. There was a great increase in the potatoe disease on August 30th.

*Alford.*—Dr. West writes: "We have had a great deal of remittent fever and typhus throughout the quarter; during the latter weeks more or less complicated with diarrhœa and sore throat. The autumnal diarrhœa will now, probably, take the place of the fevers, which, caused probably by malarious influences during the late dry season, when all drains are stagnant pools instead of being running streams, will, now that rain has at length come, gradually, it is hoped, leave us altogether. Diphtheria, too, which had been frequently, in a slight form, a complication of some of the fever cases, seems likely to assume the dignity of a distinct malady; while the epidemic influence which has caused it, will probably bring us a supply of its cognate disease, scarlatina, to take its place. Hooping-cough, followed in many instances by measles, has prevailed during the whole of the quarter."

*Gainsborough.*—Dr. Mackinder says: "We have just passed over what may fairly be considered the most healthy quarter of the year. The most common diseases have been hooping-cough and affections of the air-passages. Diarrhœa has been much less frequent than usual, and generally of a bilious character, with colicky pains; though we have had a few cases of a more aggravated nature, with choleraic purging, cramps, and slight collapse. One, on the 22nd of August, had suppression of urine for twelve hours. On the 18th of June all the boys, family, and servants, in a large school were attacked with diarrhœa, before 6 A.M., presumedly from suppressed cutaneous exhalation, the thermometer having run down thirteen degrees in forty-eight hours. All recovered quickly with domestic treatment. Small-pox has been again introduced. The first case occurred on July 24th. Three weeks previously, in consequence of the sudden death of her sister (the mother of the child first seized), a young woman came from Walesby,—a village about seventeen miles hence,—where the small-pox was prevalent. She had had it badly, and had left a sister suffering from the same disease, neither of them having been vaccinated. This person states that nearly all the affected were adults. Those who had been vaccinated had it mildly, those unvaccinated severely, and some paid the penalty of parental neglect by a forfeiture of their lives. Thus then, after an incubation of three weeks, the infection having been conveyed by a person who had been afflicted with the disease a month before, the small-pox first manifested itself in a little child seven years old, who had been vaccinated. It was a good specimen of the discrete form. Another vaccinated child in the same family sickened, but escaped; while a third member, whom the kind father would not allow to be vaccinated, passed through the various stages of the malady. Several children in the neighbourhood have been affected; the vaccinated ones had it very mildly, some having only a few pocks; the unvaccinated had it severely, one case being confluent. We have now a case under treatment. It is high time more vigorous measures were taken to prevent the extension of this formidable disease. A period ought to be fixed, before which no one convalescing from small-pox ought to be permitted, however severe it may seem, to go abroad. Certainly not with the highly tinted evidence of variolous pustulation impressed, like a brand of dishonour, on every square inch of the exposed surface of the body. This is the second time small-pox has been introduced into Gainsborough by imprudent and morally censurable people. As in the former invasion, so in this, I hope, preventive measures, vaccination and seclusion, will soon prove an effectual barrier to the progress of the intruder. A haymaker fell down from sunstroke a few minutes before ten on the morning of June 22nd. The sky was unobscured, and the thermometer standing at 112°. He recovered, and resumed his work in the course of a day or two, but is now suffering from suspicious cephalic disease. One woman died suddenly, seventeen days after an ordinary labour, from presumed occlusion of some arterial or venous channel. There were no premonitory symptoms; she was *in extremis*

when seen; a *post mortem* examination was not allowed. Another woman had a severe attack of puerperal mania, forty-seven days after a normal confinement. She did well. A third died of intrauterine hæmorrhage at the eighth and half month from conception. When first seen she was in a state of icy collapse, from which she never rallied. No external bleeding. The *post mortem* examination revealed an absolutely bloodless condition of the whole body and viscera; but within the uterus, and between the decidua, was an immense clot of blood, forming a thick stratum on the parental surface of the placenta. The decidua formed a *cul-de-sac* from which not a single drop of blood had escaped. This poor woman had had a large family, and had been half-starved for some time, but would not confess her poverty. Her enfeebled state was, doubtless, the predisposing, and the lifting of a heavy weight the exciting cause of her sudden death. One child died suddenly from epileptoid convulsions. The necropsy showed recent and remote endocranial mischief, with a highly congested condition of the kidneys, liver, and pancreas. We have still a few cases of throat affection, all of a mild character and soon relieved by appropriate treatment. Eighty-seven paupers were attended as fresh cases during the quarter, thirty-two in June, twenty-eight in July, and twenty-seven in August. Of these, eleven were between 60 and 70 years old; twelve between 70 and 80; and four between 80 and 90 years old. Two females had each attained the age of 89. One of them died of diarrhœa, and the other is recovering from a fracture of the neck of the thigh-bone; an accident of common occurrence here, and one that generally does well, the sufferers regaining the full powers of their limbs. One hundred and seventy-three pauper cases were attended in the quarter ending May 28; *minus* one of double the number that have been attended this past quarter.

"Herewith, I have sent an account of the births and deaths of the quarter, for the township of Gainsborough, kindly granted by the registrar, Mr. Brownlow." (See p. 311.)

The subjoined meteorological report has been furnished by Mr. Dyson:—"The barometric pressure during the month of June, till the 19th, was variable, and storms of thunder, lightning and rain, were frequent; the remainder of the month was unceasingly fine. Wheat was in ear as early as the 8th, and in flower on the 13th, nearly a fortnight sooner than the average. The first wheat was cut on the 26th of July, and the harvest became general immediately afterwards; very nearly the whole of the abundant wheat-crop was housed before the 1st of September, and in fine condition. Very fine weather prevailed throughout July and August. The temperature reached 92° on the 16th of June, 85° on the 24th of July, and 89° on the 12th of August. The coldest nights were 41° on the 27th of June, 43° the 19th, 29th, and 31st of July, and 45° on the 25th of August. The average fall of rain in the year, is 24 inches, and very little more than 6 inches had fallen in the first six months. In June, 1·54 inch fell; in July, 2·62; and in August, 2·42, of which more than one inch fell in the night of the 18th. Generally, the weather during the quarter has been unusually fine, and all cereal crops were most prolific; but the bean-crop was, generally, a complete failure."

*Warrington.*—Mr. Spinks states that the season has been unusually healthy.

*Wigan.*—Mr. Hussey writes: "Measles and hooping-cough have prevailed to as great an extent in this locality of late as scarlet fever did after the last cholera epidemic. The mortality in both has been very marked, owing to the little patients yielding to inflammatory chest disease in the former and head symptoms in the latter; for the most part, however, through neglect in not seeking timely advice. The most remarkable feature in these diseases is, that a great number of the children laboured under both, *i.e.*, measles and hooping-cough, at one and the same time, the latter proving fatal in proportion to the severity of the former: in fact, in severity they have gone hand in hand. And the measly eruption, I have noticed, did not prove, as it does when the disease is uncomplicated, *a full crop.* One of my juvenile patients (vaccinated by myself two years ago) passed into varioloid out of a severe attack of simple scarlatina, in which the arms and forehead were studded with large miliary vesicles. So large, indeed, were the latter, that at first sight I mistook them for drops of sweat. Diarrhœa of a severe character, with vomiting and cramps, and dejections rice-water like, has been very common of late; and, for the most part, I could not trace the disease to errors in eating or drinking. Its recurrence this season has dated from the beginning of the last change in the weather, which has been cloudy, with occasional heavy rains. Wind principally from the north. The sickening emanations from cesspools during the heat of the last quarter, as evidenced by the sense of smell, have had much to do, I fear, with infantile diseases. Query, has the emptying of cesspools in the night time, when people are asleep, any material bearing upon the propagation of disease? Close by one of these, a woman lately died of phthisis; a child in the same house is fearfully scrofulous; and a young man, a patient of mine in the next house, is at the present time likely to die of phthisis."

*Township of Gainsborough (population in* 1851, 7,261). *Deaths from May* 29*th to August* 27*th,* 1858, *inclusive.*

| Date. | Age. | Sex. M. | Sex. F. | Occupation. | Cause of Death. |
|---|---|---|---|---|---|
| My.30 | 1 yr. | 1 | — | S. of joiner | Pertussis |
| June1 | 3 wks. | 1 | — | S. of domestic servant | Marasmus |
| „ | 1 yr. | 1 | — | S. of cordwainer | Pertussis |
| 4 | 50 „ | — | 1 | W. of publican | Softening of the brain |
| 7 | 56 „ | — | 1 | W. of labourer | Disease of liver, jaundice |
| 10 | 1 „ | — | 1 | D. of tanner | Pertussis, pneumonia |
| 13 | 5 „ | 1 | — | S. of publican | Bronchitis, pertussis, tabes mesenterica |
| 14 | 52 „ | 1 | — | Labourer | Phthisis |
| 15 | 41 „ | — | 1 | W. of butcher | Carcinoma of stomach |
| 16 | 76 „ | 1 | — | Tailor | Atonic apoplexy |
| „ | 50 „ | — | 1 | W. of ironmonger | Cancer uteri |
| 21 | 86 „ | — | 1 | W. of joiner | Paraplegia |
| 22 | 1 „ | — | 1 | D. of joiner | Pertussis, dysenteria |
| „ | 63 „ | — | 1 | W. of labourer | Bronchitis |
| 23 | 1 „ | 1 | — | S. of single woman | Manslaughter* |
| 27 | 10 mo | — | 1 | D. of labourer | Decline from birth† |
| 28 | 8 „ | — | 1 | D. of blacksmith | Bronchitis, pertussis |
| 29 | 9 wks | 1 | — | S. of dressmaker | Sec. syphils, cong. of right lung, granular disease of kidney‡ |
| July 3 | 6 „ | 1 | — | S. of merchant | Collapse of the lung |
| 4 | 19 yrs | 1 | — | Tailor | Phthisis |
| „ | 29 „ | — | 1 | W. of staymaker | Hyperlactation & mental emotion, presumed clot in heart |
| „ | 82 „ | 1 | — | Cordwainer | Diarrhœa |
| 5 | 69 „ | — | 1 | Dressmaker | Paraplegia |
| 6 | 6 „ | — | 1 | D. of joiner | Pneumonia, dysentery |
| 7 | 71 „ | — | 1 | W. of seaman | Fracture of femur, bedsore, etc. |
| 10 | 52 „ | — | 1 | W. of labourer | Diarrhœa |
| 11 | 81 „ | — | 1 | W. of cottager | Disease of heart |
| 13 | 2 „ | 1 | — | S. of domestic servant | Bronchitis |
| 17 | 8 mo | — | 1 | D. of waterman | „ |
| 20 | 90 yrs | — | 1 | W. of coal-porter | Diarrhœa |
| 21 | 73 „ | 1 | — | Weaver | Valvular dis. of heart, diarrhœa |
| 23 | 3 „ | — | 1 | D. of boatman | Meningitis |
| 27 | 73 „ | — | 1 | W. of cordwainer | Old age† |
| 28 | 5 wks | — | 1 | D. of staymaker | Inanition, dysentery |
| „ | 3 „ | — | 1 | D. of labourer | Convulsions |
| 29 | 1 yr. | 1 | — | S. of waterman | Pertussis, tabes mesenterica |
| Aug.3 | 88 „ | — | 1 | W. of labourer | Decay of nature |
| 4 | 10 dys | 1 | — | S. of baker | Premature birth |
| 8 | 77 yrs | — | 1 | W. of coal-measurer | Natural decay |
| 9 | 33 „ | 1 | — | Whitesmith | Phthisis |
| 10 | 55 „ | 1 | — | Retired joiner | Gangrena senilis |
| 11 | 6 dys | 1 | — | S. of woollen draper | Premature birth |
| 12 | 57 yrs | 1 | — | Innkeeper | Gastro-enteritis |
| „ | 3 mo | 1 | — | S. of carpenter | Marasmus |
| 13 | 7 yrs | — | 1 | D. of cabinet maker | Encephalitis |
| 16 | 8 „ | — | 1 | D. of seaman | Epileptoid convulsions, congestion of kidneys, arachnoid membrane with old adhesions‡ |
| 19 | 40 „ | — | 1 | W. of general dealer | Intrauterine hæmorrhage‡ |
| 20 | 80 „ | 1 | — | Labourer | Bronchitis senilis |
| 21 | 19 „ | — | 1 | Domestic servant | Phthisis |
| 27 | 3 wks | 1 | — | S. of pork butcher | Atrophy |
| Total .... | | 22 | 28 | = 50 | |

* Certified by coroner. † Cause of death not certified. ‡ Certified after *post mortem.*

Two of the deaths and three of the births occurred in the workhouse. The number of births during the same period were 28; viz. males 16, females 12. The total of births registered in the quarter is much below the average.

*Gainsborough Quarterly Medical and Meteorological Register.*

| Treatment commenced. | Diseases of Private Patients. | Diseases of Pauper Patients. | Barometric pressure at the temp. of 32° Fahr. | Temperat. Dry bulb. | Temperat. Wet bulb. | Direction of Wind. | Rain in 24 hours. | Ozone. (Schoen.) | Remarks. |
|---|---|---|---|---|---|---|---|---|---|
| May 29 | Pertussis, bronchitis, emphysema | Phthisis, lumbago | 30·088 | 56 | 52 | SW. | ·11 | — | Clear |
| 30 | Sclerotitis, tabes mesent., hoop.cough, tonsillitis, bronchitis | | 30·012 | 69 | 60 | W. | ·0 | 2 | „ |
| 31 | Hoop.cogh., diar., bronchitis, stomatitis [ulcerosa | Phthisis, amenorrhœa | — | 73 | 64 | S. | ·0 | — | „ |
| June 1 | Dyspepsia, bronchitis | Catarrh, bronchitis, amenorrhœa | 30·028 | 70 | 62 | W. | ·0 | — | „ |
| 2 | Diarrhœa | Amenorrhœa, eczema impetiginoides | 30·079 | 68 | 62 | NW. | ·59 | 3 | Thun., lg., & [rain |
| 3 | Catarrh, gastritis | Hæmaturia, diarrhœa, anasarca | 29·949 | 63 | 62 | SE. | ·02 | 4 | Fine |
| 4 | | Catarrh, anasarca | 30·052 | 67 | 58 | SW. | ·0 | — | „ |
| 5 | Dysmenorrhœa, pemphigus, leucorrh. | Scabies, colic | 30·087 | 59 | 56 | S. NW. | ·0 | — | „ |
| 6 | Vermes | | 30·145 | 68 | 59 | NW. N. | ·01 | — | Thunder |
| 7 | Neuralgia | Diseased heart, incipient paralysis | 30·169 | 57 | 54 | NE. | ·0 | 5 | Fog |
| 8 | | Catarrh, colic, hæmorrhoids | 29·989 | 65 | 48 | E. | ·0 | 5 | Wheat in ear |
| 9 | | Colic, stomatitis, dyspepsia, catarrh | 29·906 | 64 | 63 | N. | ·16 | 10 | Thund. & lg. |
| 10 | Urticaria, diarrhœa | Faucitis, bronchitis, phthisis | 30·109 | 68 | 61 | N. | ·0 | 2 | Fine |
| 11 | Neuralgia | Curvature of spine, neuralgia | 30·024 | 68 | 61 | NE. | ·0 | 7 | „ |
| 12 | Hæmorrhoids, catarrh, bronchitis, diarrhœa with cramps | Neuralgia | 29·960 | 59 | 57 | E. | ·44 | 10 | Thn., lg. & rn. |
| 13 | Hooping cough, urticaria, dysentery, diarrhœa, dyspepsia | Colic | 29·905 | 69 | 64 | S. | ·04 | 10 | Fine, wheat in flower |
| 14 | Pleurodynia, bronchocele, hæmorrhoids, cramps from hyperlactation | Paralysis, phlegmasia | 29·856 | 74 | 68 | SE. | ·0 | 4 | „ |
| 15 | | Syphilis | 29·984 | 79 | 69 | S. | ·0 | 3 | „ |
| 16 | Colic, bronchocele | Bronchitis, hæmorrhoids | 29·932 | 80 | 71 | N. | ·0 | 6 | Dist. thund. |
| 17 | | Syphilis, adenitis, diarrhœa | 29·702 | 72 | 68 | N. | ·09 | — | Fine |
| 18 | Dysentery, diarrhœa | | 29·979 | 63 | 58 | N. | ·0 | 4 | „ |
| 19 | Stomatitis | Pityriasis, dysentery | 30·053 | 72 | 61 | NW. SW. | ·20 | — | Fog, fine |
| 20 | Conjunctivitis, measles, gingivitis | | 30·096 | 72 | 62 | NW. | ·0 | 10 | Fine |
| 21 | Neural., congestio cerebri, diar., dysen. | Adenitis, carbuncle | 30·268 | 69 | 62 | NE. | ·0 | — | Fog, fine |
| 22 | Sun stroke, parotitis, aphtha | Eczema impetiginoides | — | — | — | — | ·0 | — | Clear to end |
| 23 | Menorrhagia | Bronchitis, diarr., abscess of abdomen | — | — | — | — | ·0 | — | of month |
| 24 | Herpes, menorrhagia, colic, scarlatina | Pleurodynia | — | — | — | — | ·0 | — | |
| 25 | Scabies | | — | — | — | — | ·0 | — | |

| | | | | | | | | | |
|---|---|---|---|---|---|---|---|---|---|
| 26 | | Acne | — | — | — | — | ·0 | — | |
| 27 | Diarrhœa, hooping cough | | — | — | — | — | ·0 | 4 | |
| 28 | Hyperlactation, psoriasis, paralysis | | — | — | — | — | — | — | |
| 29 | | Pleurodynia, diarrhœa | — | — | — | — | — | — | |
| 30 | Diphtheria | Synovitis | — | — | — | — | — | — | |
| July 1 | Amenorrhœa, furuncle, scabies | | 30·065 | 61 | 52 | NW. | ·0 | — | |
| 2 | | | — | 59 | 52 | NW. | ·0 | — | |
| 3 | Hooping cough | | — | — | — | — | ·0 | — | |
| 4 | Hooping cough, colic | | — | — | — | — | ·05 | — | |
| 5 | Diarr., dyspepsia, gravel, pleurodynia | | — | — | — | — | ·0 | — | |
| 6 | Uterine hæmorrhage, pleurodynia, emphysema, bronchitis | Vertigo, diarrhœa | — | — | — | — | ·0 | — | |
| 7 | Orchitis, catarrh, menorrhagia, diarr. | Diarrhœa, parotitis | — | — | — | NW. | ·03 | 10 | Fine |
| 8 | Paralysis, diarr., lumbago, psoriasis, | | 29·760 | 55 | 54 | NW. | ·54 | 10 | Heavy rain |
| 9 | Ophthalmia, epistaxis [neuralgia | Aphtha | 29·937 | 58 | 53 | NE. | ·01 | 10 | Fine to 24th |
| 10 | Corneitis, œdema, hæmorrhoids, scar- | | 29·940 | 63 | 56 | N. | ·0 | 10 | |
| 11 | Pleurodynia [latina, varicella | Colic | 30·083 | 72 | 60 | N. | ·11 | — | |
| 12 | Pleurodynia, colic | Bronchitis | 29·913 | 68 | 64 | S. | ·0 | — | |
| 13 | Anæmia, faucitis, amenor., emphysema | | 29·885 | 75 | 65 | S. | — | — | |
| 14 | Dyspepsia, diarrhœa, hæmorrhoids | Dentitis, imbecility, colic | — | 68 | 64 | S. | ·14 | — | |
| 15 | Intercostal neuralgia, faucitis | | 29·881 | — | — | — | — | — | |
| 16 | Febricula | Diarrhœa, scabies, morbus coxarius | — | — | — | ch. | ·92 | — | |
| 17 | Dyspepsia | Rupia | 29·863 | 64 | 60 | N. | ·01 | 10 | |
| 18 | Cataract | Insanity | 30·063 | 66 | 61 | N. | ·04 | — | |
| 19 | Diarrhœa with vomiting, colic | Lumbago, erysipelas, diarrhœa | 30·160 | 68 | 59 | W. | ·0[illegible] | — | |
| 20 | Faucitis, purulent ophthalmia | Neuralgia, rheumatism | 29·971 | 73 | 62 | NE. SE. | ·33 | 10 | |
| 21 | Dysentery | Anæmia | 29·729 | 59 | 57 | SE. W. | ·0 | — | |
| 22 | Pleurodynia | | 29·870 | 65 | 59 | SW. | ·01 | — | |
| 23 | | Pleurodynia, strumous ophthalmia | 29·729 | 72 | 61 | SW. | ·03 | — | [rain |
| 24 | Variola | | 29·771 | 72 | 63 | SW. W. | ·18 | — | Boisterous w. |
| 25 | | Dysentery, diarrhœa, anasarca | 29·225 | 53 | 52 | SE. W. | ·22 | — | Clear and |
| 26 | Faucitis, rheumatism, parotitis | Periostitis | 29·801 | 63 | 54 | W. | ·0 | — | fine to Au- |
| 27 | Menorrhagia, enlarged liver | Anasarca, hydrops | 29·855 | 63 | 58 | NE. | ·0 | — | gust 10th |
| 28 | Conjunctivitis | Choleraic diarrhœa | 29·845 | 62 | 55 | NE. | ·0 | — | |
| 29 | Amenorrhœa, catarrh | Congestio cerebri | 29·970 | 62 | 56 | NE. | ·0 | — | |
| 30 | | Diarrhœa, ascites | 30·000 | 64 | 57 | N. | ·0 | — | |
| 31 | Dyspepsia | | 30·153 | 66 | 57 | NW. | ·0 | — | |

| Treatment commenced. | Diseases of Private Patients. | Diseases of Pauper Patients. | Barometric pressure at the temp. of 32° Fahr. | Temperat. Dry bulb. | Temperat. Wet bulb. | Direction of Wind. | Rain in 24 hours. | Ozone. (Schoen.) | Remarks. |
|---|---|---|---|---|---|---|---|---|---|
| Aug. 1 | Abortio | | 30·187 | 67 | 58 | N. | ·0 | 8 | |
| 2 | Leucorrhœa [pepsia | Rheumatism | 30·044 | 63 | 57 | E. | ·0 | — | |
| 3 | Colic, valvular disease of heart, dys- | Puerperal mania | 29·718 | 66 | 59 | SE. | ·0 | — | |
| 4 | Angina pectoris, faucitis, encephalitis | | 29·893 | 70 | 63 | W. SW. | ·08 | 3 | |
| 5 | Bronchitis, pustular ophthalmia, gas- | Colic, lumbago | 29·863 | 65 | 58 | W. | ·0 | — | |
| 6 | Diarrhœa [tritis, dysuria | Dyspepsia, dysuria | 30·038 | 64 | 57 | W. NW. | .01 | — | |
| 7 | „ , congestio mucosiæ, poisoning by prussic acid in the process of photography | | 30·368 | 69 | 58 | N. ch. | ·0 | — | |
| 8 | Gastrodynia, phthisis | Bronchitis | 30·461 | 74 | 62 | NE. | ·0 | — | |
| 9 | Ulcer of bowel, pleurodynia, scarlatina, | Dyspepsia, bronchitis | 30·297 | 65 | 59 | E. | ·0 | 3 | |
| 10 | Ulcus uteri [febricula | Colic, catarrh | 30·132 | 65 | 60 | NE. | ·0 | 2 | |
| 11 | Sciatica, encephalitis, febris, hooping cough, anasarca | Adenitis, emphysema | 30·057 | 63 | 60 | NE. | ·10 | 5 | Rain |
| 12 | Diarrhœa, stomatitis | Catarrh | 30·003 | 67 | 64 | NE. | ·14 | — | Thun., lg., & heavy rain |
| 13 | | Leucorrhœa, congestio cerebri, syphilis | 29·949 | 64 | 60 | NW. | ·53 | — | |
| 14 | | Diarrhœa | 29·967 | 62 | 58 | — | ·0 | — | Fine to 22nd |
| 15 | Faucitis, hooping cough | | 29·919 | 68 | 60 | NW. | ·0 | 6 | |
| 16 | Meningitis, nephritis, anæmia, anasarca | Syphilis, inflamed absorbents of arm | 29·970 | 70 | 63 | S. | ·0 | 8 | |
| 17 | Neuralgia, faucitis, dysuria, bronchitis, hooping cough | Amenorrhœa, rheumatism, diarrhœa, English cholera | 29·802 | 70 | 62 | SE. | ·02 | 10 | |
| 18 | Diarrhœa, carbuncle, variola, intra-uterine hæmorrhage | Rheumatism, vertigo | 29·719 | 69 | 62 | SE. | 1·03 | 10 | |
| 19 | English cholera, hooping cough, faucitis, pustular ophthalmia | Hooping cough | 29·605 | 72 | 68 | NE. | ·0 | — | |
| 20 | Cellulitis, menorrhagia, epistaxis | Hooping cough, gonorrhœa | 29·761 | 62 | 59 | W. | ·04 | — | |
| 21 | Faucitis, diarrhœa, cephalagia, dentitis | Stomatitis, lumbago | 29·810 | 58 | 54 | NW. | ·29 | 10 | |
| 22 | „ , colic | Diarrhœa, English cholera | 29·895 | 65 | 63 | NW. | ·0 | 7 | |
| 23 | | Prurigo | 30·027 | 65 | 61 | NE. | ·0 | — | Fog, fine |
| 24 | Leucorrhœa, anasarca | Erysipelas | 30·110 | 65 | 62 | NW. | ·0 | — | „ „ |
| 25 | Hæmorrhoids | | 30·012 | 62 | 58 | NW. | ·0 | 4 | Fine |
| 26 | Ophthalmia, colic, English cholera | Bronchitis | 30·059 | 55 | 49 | NW. | ·04 | — | „ |
| 27 | Faucitis, glossitis, diarrhœa | Morbus coxarius, tænia | 29·832 | 62 | 55 | NW. | ·04 | — | „ |

*Bolton-le-Moors.*—Mr. Pendlebury writes: "The past quarter has not been a very sickly one on the whole. Small-pox has been (comparatively) the most prevalent disease, and has in a marked manner ferreted out the unvaccinated portion of families, not sparing the adults. Cases of summer cholera have not exceeded the average number. Diarrhœa and dysentery have only been moderately prevalent. The summer has been remarkably fine and dry, with no long-continued heat."

*York.*—Mr. Procter writes: "The health has, in this place, during the quarter, been generally good. Eruptive fevers have been prevalent, but not to an unusual extent. Diarrhœal cases have not been so prevalent as is ordinarily the case at this time of year; and they, as well as the few cases of dysentery which have appeared, have been slight and easy of management."

*Rothbury.*—Mr. Summers says: "That two children, suffering from severe hooping-cough, came to this place from Newcastle, and remained here five weeks, mixing quite freely with other children; but without communicating the disease in any instance."

*Lerwick.*—Dr. Spence writes: "With the exception of the last week, which was rainy, the month of June was unusually fine; and during a fortnight the weather was warmer than had been known in Shetland for some years. The prevailing wind was from the S.W. July was not so fine, being more changeable, with rain, fog, and high winds. The winds were chiefly S.W., but easterly and northerly winds also prevailed. The first fortnight of August was fine, with south-westerly winds: in the last fortnight the weather was changeable, with much rain and fog, and the winds were S., S.E., N., and N.W. The average development of ozone was in June 7.5, in July 6.3, and in August 6.2. In the end of last quarter, and the beginning of this, there were twenty cases of small-pox, exclusive of the man who introduced it: of these ten were unvaccinated; and one death occurred in a boy who was said to have been vaccinated. Measles were imported from Edinburgh in the month of August; only three cases occurred, and they were very slight. There was less than the usual amount of sickness of any kind during the quarter. Ten funerals took place during the quarter, but of these, two were of sailors who died on shipboard; one was of a man who was drowned, and another of a man who was killed by an accidental explosion in blasting. Of the remaining six, the average age at death was 35; one being above 70, and one below 5. The causes were: small-pox, 1; chicken-pox, 1; phthisis, 1; hectic from diseased bone, 1; cancer of the stomach, 1; and debility from old age, 1.

